# Spectrum of IDH-mutant tumors in Ollier-Maffucci disease: the triple interaction theory

**DOI:** 10.1186/s13023-024-03457-7

**Published:** 2024-11-25

**Authors:** Emmanuel Mandonnet, Thomas Funck-Brentano, Jean-Philippe Hugnot, Mehdi Touat

**Affiliations:** 1 Department of Neurosurgery, Lariboisière Hospital, AP-HP, 2 rue Ambroise Paré, Paris, 75010 France; 2 Frontlab, CNRS UMR 7225, INSERM U1127, Paris Brain Institute (ICM), Paris, France; 3 Université Paris Cité, Paris, France; 4 Department of Rheumatology, Lariboisière Hospital, APHP.Nord, Université Paris Cité, Paris, France; 5 Université Paris Cité, Inserm U1132, BIOSCAR, Paris, F-75010 France; 6 Institut de Génomique Fonctionnelle, Université de Montpellier, CNRS, INSERM, Montpellier, 34091 France; 7 Sorbonne Université, Inserm, CNRS, UMR S 1127, Institut du Cerveau, ICM, AP-HP, Hôpitaux Universitaires La Pitié Salpêtrière - Charles Foix, Service de Neuro-oncologie, Paris, France; 8 Department of Neurology, Brigham and Women’s Hospital, Boston, USA

**Keywords:** Ollier-Maffucci, IDH mutation, Glioma, Single nucleotide polymorphism, Tumors

## Abstract

We propose to refine our understanding of the pathophysiology underlying the tumor spectrum observed in patients with Ollier disease (OD) and Maffucci syndrome (MS). On one hand, assuming that all IDH-mutated tumors (as well as enchondromas) observed in OD-MS patients derive from one IDH-mutant cell giving rise to different lineages, the observation of different tumors arising in organs deriving from the neuroectoderm, mesoderm and endoderm points towards a very early post-zygotic event for the IDH mutation. To explain then that the spectrum of IDH-mutated tumors is restricted to some types of tumors, we propose the following hypothesis: - First, we posit that not every mutated cell of the lineage will “express” the IDH mutant phenotype. This can be due i/ to the disappearance in some tissue of the IDH-mutated clone due to negative selection pressure later in embryo development ii/ to the lack of expression of the IDH1 protein in specific cell types iii/ to a functional cell state not leading to the accumulation of the oncometabolite D-2-hydroxyglutarate (D-2HG) in that tissue/organ. - Second, generalizing the recent understanding of the gliomagenesis in the general population bearing the rs55705857 G-allele variant at 8q24.21, we postulate that OD-MS patients with an inheritable predisposing single nucleotide polymorphism (SNP) are more likely to develop a malignancy, with a specific SNP for each kind of tumor/organ. In summary, our theory provides a new understanding of IDH-mutated tumors in OD-MS patients, as arising from the triple interaction within the same cell of a developmental defect (the somatic mutation that occurs early during the embryogenesis), an organ-specific functional state “expressing” the IDH mutation and leading to an accumulation of D-2HG, and an inheritable predisposing factor (a risky SNP, also specific to each organ). We discuss how this theory could guide future research in OD-MS patients and, more generally, in patients harboring sporadic IDH-mutated tumors.

**Supplementary Information:**

The online version contains supplementary material available at 10.1186/s13023-024-03457-7.

Ollier disease (OD) and Maffucci syndrome (MS) are exceptionally rare conditions, characterized by multiple enchondromas involving mainly the limbs, with additional soft tissue hemangiomas for MS. A major step in the understanding of these diseases has been achieved in 2011, with the discovery of somatic mosaic mutations in isocitrate dehydrogenase 1 or 2 genes (therefore referred as IDH mutations) in OD and MS patients with enchondromas and hemangiomas [1, 2]. It has been recognized over the years that OD-MS predispose to multiple types of malignancies during the course of the disease [3]. In particular, increased incidence of glioma [4], cholangiocarcinoma [5], acute myeloid leukemia [6], and ovarian juvenile granulosa tumor [7] have been reported. Interestingly, in all cases for which paired tumor samples were available, the same IDH mutation was found in the enchondromas and in the non-cartilaginous tumors [7]. Moreover, to the notable exception of the ovarian juvenile granulosa tumor, all these tumors are also the most frequent IDH-mutated tumors arising sporadically (i.e. in absence of OD-MS).

Assuming that all IDH-mutated tumors (as well as enchondromas) observed in OD-MS patients derive from one IDH-mutant cell giving rise to different lineages, the observation of different tumors arising in organs deriving from the neuroectoderm (glioma), mesoderm (blood, cartilage) and endoderm (liver) points towards a very early post-zygotic event for the IDH mutation.

But if the mutation can be found ubiquitously in all kinds of tissue, the following question arises: why do we not observe IDH-mutated tumors of some tissue/organs (in OD-MS patients as well as sporadically), such as melanoma or schwannoma, despite that the mosaicism should also be found in melanocytes or Schwann cells? Two mechanisms could be at stake:

- The mosaicism could be lost in some cell lineage, due to negative pressure selection [8]. Under this hypothesis, the IDH mutation would not be found in some specific tissue, like the melanocytes of the skin or the Schwann cells of peripheral nerves.
- Alternatively, the IDH mutation might not be “expressed” in all tissues. Indeed, depending on the functional state of the cell (and especially its metabolism), some cells might not produce D-2-HG, or not in sufficient quantity to induce substantial epigenetic remodeling, which is the first step of IDH-mutation mediated oncogenesis [9–11]. The IDH protein may also be not expressed in certain tissues, thereby sparing them from manifesting the mutant phenotype.

However, if the IDH mutation is an early post-zygotic event, one might wonder why not every OD-MS patient will develop the whole spectrum of IDH-mutated malignancy.

The response might lie in the recent understanding at the molecular mechanistic level of IDH-mutated gliomagenesis. Researchers elucidated the mechanisms underlying the increased risk of developing an IDH-mutated glioma in people bearing the rs55705857 G-allele variant at 8q24.21 [12, 13]. This allele resides in an intron of the long noncoding RNA CCDC26. It has been demonstrated that the rs55705857 variant produces a brain-specific oncogenic activity in IDH-mutated tissue, through an epigenetically-mediated dysregulation of the MYC gene [14, 15]. Consequently, the frequency of the G-allele risky variant should correlate with the frequency of glioma in OD-MS patients: we would expect higher risk of glioma in Scandinavian (G-allele frequency of 13%) than in Turkish (G-allele frequency of 1%) OD-MS patients [16].

Extrapolating a similar mechanism for tumors from other tissues, one can postulate that from a dispersed and large contingent of IDH-mutated cells derived from a common cell lineage, only those patients bearing other tissue-associated predisposing genomic variants will develop a tumor, with a molecular mechanism specific to each organ microenvironment. This is the same reasoning that was recently proposed to explain the enchondromatosis itself in OD-MS patients: germline (or early post-zygotic) variants were evidenced in genes of the HIF-1α pathway [17].

There is of course an alternative explanation to the absence of tumor development in some OD-MS patients: the mutation might have occurred at a later stage, and thus would have targeted only cartilaginous tissue.

In summary, we propose that IDH-tumors in OD/MS patients arise from a triple hit mechanism:

- A somatic mosaic IDH mutation occurring early during embryogenesis in most patients,
- An organ-specific functional state cell “expressing” the mutation, resulting in an accumulation of D-2-HG, triggering the second event of epigenetic reconfiguration of DNA,
- A predisposing organ-specific SNP variant, which will foster the initiation of proliferation in the epigenetically reconfigured cell.

Such advances open new avenues. First, we would like to emphasize the importance of post-mortem tissue analysis. As the IDH-mutation cannot currently be analyzed non-invasively in the different types of tissue, post-mortem autopsy is the only way to determine the ubiquitous nature (or not) of the IDH-mutation in OD-MS patients.

Secondly, it is essential to screen for the rs55705857 G-allele in every patient diagnosed with OD-MS. Indeed, we predict that the risk of developing a glioma if the variant is detected should be close to 100% (under the hypothesis of ubiquitous IDH-mutation). If confirmed, this finding would enable personalized follow-up care, such as scheduling regular brain MRIs [18] specifically for patients carrying the high-risk variants.

Conversely, we could leverage the OD-MS population for identifying new variants at risks for each tumor of the whole spectrum of IDH-mutated tumors, by comparing SNPs in OD-MS patients with versus without a specific tumor.

Lastly, due the extreme rarity of this clinical entity, an international effort should be initiated to build a well-annotated patient cohort, comprising comprehensive clinical, radiological, and molecular data.

## Electronic supplementary material

Below is the link to the electronic supplementary material.

Supplementary Material 1

Supplementary Material 2

## Data Availability

NA.

## Abbreviations

SNP: Single Nucleotide Polymorphism
OD: Ollier Disease
MS: Maffucci Syndrome
IDH: Isocitrate Dehydrogenase
