# Supplementary material for: Spectrum of IDH-mutant tumors in Ollier-Maffucci disease: the triple interaction theory
Source: Orphanet J Rare Dis. 2024 Nov 25;19:434. doi: 10.1186/s13023-024-03457-7 (PMC11587773; doi:10.1186/s13023-024-03457-7)
Supplement: Supplementary file 1 — Supplementary Material 1 [file 13023_2024_3457_MOESM1_ESM.docx]

Reviewer #1: The authors aim to propose a new theory regarding the development of gliomas (and more generally neoplasms) in OD/MS patients. Although I agree that OD/MS is a particularly interesting pathology to understand the gliomageneis of IDH mutant tumors I am not completely convinced by the authors arguments.

Response: We thank the reviewer for the thoughtful comments. We fully agree that our theory suffered from some inconsistencies. In the revised version, we rather hypothesize that the somatic mosaicism occurs very early in the embryogenesis, as the tumors observed in OD-MS patients develop in tissue/organs deriving from the neuroectoderm, mesoderm, and endoderm. Consequently, we gave up the NCSC theory and reframed the letter to focus on the pathophysiology of the IDH-mutated tumors seen in some (but not all) OD-MS patients.

Major comments:
- since the authors aim to propose a new theory about the MS/OD pathophysiology they should explicitely state to which (old) theory their theory is opposed to

Response: We propose a new theory regarding the pathophysiology of the IDH-mutated tumors observed in OD-MS patients. To the best of our knowledge, any theory on this topic has never been proposed before.

- based on the authors' knowledge, is it really possible based on the analysis of affected tissues to identify the cell in which mutations arise in the context of somatic mosaicism?

Response: We agree that we cannot identify the cell in which mutations arise in the context of somatic mosaicism based on our current knowledge. We now mention in the revised version that currently, only post-mortem analysis would enable to determine the ubiquitous nature (or not) of the IDH mutation in the different types of human tissue.

- to the best of our knowledge, neural crest stem cells give rise to neurons and glia from the peripheral nervous system. How does this fit with the development of gliomas, i.e CNS tumors ?

Response. We fully agree, and as already explained above, we gave up the NCSC theory.

- therefore in the abstract the sentence: "We first demonstrate that all IDH-mutated tumors (as well as enchondromas) develop from cells that derive from neural crest stem cells" is overstated. "Demonstrate" should be replaced by "hypothesize"

Response. We fully agree, and as already explained above, we gave up the NCSC theory.

- next, the authors state that OD/MS patients who develop gliomas should have a particular SNP (the 8q SNP at risk for IDH mutant gliomas). However, it is unclear why having this SNP should be mandatory in the context of OD/MS to develop gliomas since most patients who develop gliomas outside the OD/MS context don't require this SNP. Why would this SNP be more important OD/MS patients than in IDHmt glioma patients withour OD/MS?

Response. We fully agree, and it was our mistake. What we wanted to emphasize was that OD/MS patients with this particular SNP will almost surely develop a glioma. We modified the text accordingly.

- in order to test the second aspect of their theory the authors could genotype OD/MS patients who developed gliomas. Genotyping even a limited series of patients for the SNP at risk for gliomas would comfort the authors theory given that they speculate that the IDHmt glioma risk SNP should be present in all OD/MS patients who develop gliomas.

Response. We fully agree, but such a work would be beyond the goal of this short letter. We would be very interested to contribute to international studies to address this question.
