# Supplementary material for: Spectrum of IDH-mutant tumors in Ollier-Maffucci disease: the triple interaction theory
Source: Orphanet J Rare Dis. 2024 Nov 25;19:434. doi: 10.1186/s13023-024-03457-7 (PMC11587773; doi:10.1186/s13023-024-03457-7)
Supplement: Supplementary file 2 — Supplementary Material 2 [file 13023_2024_3457_MOESM2_ESM.doc]

To Pr Francesc Palau

Editor-in-chief of *Orphanet* *Journal of Rare Diseases*

Paris, 3^rd^ of May 2024

Dear Editor,

Please find enclosed our revised letter to the Editor entitled “Spectrum of IDH-mutant tumors in Ollier-Maffucci disease: the triple interaction theory” by Emmanuel Mandonnet, Thomas Funck-Brentano, Jean-Philippe Hugnot and Mehdi Touat.

All authors agreed to add Jean-Philippe Hugnot as a new coauthor, as his contributed significantly to this revision.

Based on the reviewer feedback, and after discussion with experts in embryology, we gave up our previous theory claiming that the mutation occurs in neural crest stem cells. Thus, in this revision, we refocused our purpose on the pathophysiology of the tumor spectrum observed in Ollier-Maffucci patients and propose a triple hit mechanism.

We thought that this could be of interest to the broad readership of *Orphanet Journal of Rare Diseases*.

We thank you in advance for considering this manuscript for publication,

Best regards,

Emmanuel Mandonnet, on behalf of all authors.

1.	Batchelor TT, Walsh KM. Understanding the Genetic Risk of IDH-Mutant Glioma. *N Engl J Med*. 2023;388(14):1332-1334. doi:10.1056/NEJMcibr2213112

2.	Yanchus C, Drucker KL, Kollmeyer TM, et al. A noncoding single-nucleotide polymorphism at 8q24 drives IDH1-mutant glioma formation. *Science*. 2022;378(6615):68-78. doi:10.1126/science.abj2890
